# Diffusion Tensor Imaging in the Diagnosis of Perianal Abscess: Protocol for a Single-Blind Randomized Controlled Trial

**DOI:** 10.2196/83449

**Published:** 2026-02-25

**Authors:** Yuting Ma, Pingping Mei, Xiutian Guo, Yan Chen

**Affiliations:** 1 Department of Anorectal, Shanghai Municipal Hospital of Traditional Chinese Medicine, Shanghai University of Traditional Chinese Medicine, Lane 68, Ronglian Road, Jiading Area, Shanghai, 201803, China, 86 15921038033

**Keywords:** diffusion tensor imaging, magnetic resonance imaging, perianal abscess, single-blind method, pelvic floor

## Abstract

**Background:**

Perianal abscesses are common anorectal conditions that often necessitate surgical intervention. Accurate preoperative assessment is crucial for effective treatment and reducing recurrence rates. Diffusion tensor imaging (DTI) is a valuable method for visualizing the degree of infection and infiltration, the extent of abscess formation, and the relationship between perianal abscess erosion. However, there is currently a lack of specific studies focusing on perianal abscesses.

**Objective:**

The objective of this study is to investigate the diagnostic utility of DTI in the preoperative assessment of perianal abscesses. By enhancing the precision of preoperative evaluation, we aim to minimize damage to the anal sphincter, reduce the recurrence rate, and improve the prognosis and quality of life for patients.

**Methods:**

This study adopts a randomized, prospective, single-blind design. Ninety participants are being randomized into 2 groups: a DTI group and a conventional magnetic resonance imaging group. A structured report is being completed based on imaging of the perianal abscesses in terms of location, number, specific pus cavity alignment, thickness, and relationship to the surrounding muscles. In addition, the patient condition is being assessed, and corresponding surgical treatment is being performed. If the patient’s blood routine shows infection, cefuroxime sodium combined with metronidazole is being administered intravenously as an anti-infective therapy. Postoperatively, the primary variable is being assessed for recurrence rate at 6 months, and the secondary variables, including postoperative pain scores on days 3 and 7, peripheral blood inflammatory factors, and assessment of anal function, are being evaluated. Normally distributed continuous data will be presented as mean (SD) and analyzed using independent or paired *t* tests. Non-normally continuous data will be analyzed with rank-sum tests. Categorical data will be expressed as frequency (%) and compared using a chi-square test or appropriate nonparametric tests. Ordinal data will be analyzed using the Ridit test. A *P* value <.05 will be considered statistically significant.

**Results:**

This study is funded by Science and Technology Commission of Shanghai Municipality Science and Technology Program (grant 23Y11920800). Patient recruitment was initiated in April 2025. As of January 2026, 37 participants have been enrolled, and data collection is scheduled to be completed in October 2026.

**Conclusions:**

DTI technique can be used to gain a deeper understanding of the relationship between internal orifice, the degree of infected infiltration, the extent of the abscess, and the involvement of the perianal tissues and muscles in patients with perianal abscess. Deep pelvic floor DTI reveals the complex 3D structure of the pelvic floor in perianal abscesses through structured reports, which may provide new insights into the diagnosis of perianal abscesses.

## Introduction

### Clinical Background and Diagnostic Challenges

Perianal abscesses are acute suppurative infections caused by obstruction of the anal gland opening and bacterial infection in the perianal tissues or spaces. They are among the most common anorectal diseases [1], particularly affecting young adults aged 20 to 40 years, with a higher prevalence in males than females [2]. Between 30% and 70% of patients with abscesses are often accompanied by anal fistula, while among patients without fistulas, the probability of developing an anal fistula is approximately 30% to 50% within months to years after abscess drainage [3]. Reducing recurrence rate and fistula rate of perianal abscesses remains a significant challenge. The recurrence rate after surgical intervention remains substantial, underscoring the need for improved diagnostic and therapeutic strategies [4].

The initial diagnosis of perianal abscesses is often based on clinical symptoms and anal palpation. However, this approach has limitations in dealing with multiple, high-grade perianal abscesses. In such cases, preoperative imaging, particularly magnetic resonance imaging (MRI), plays a pivotal role in accurate diagnosis and treatment [5,6]. MRI has a sensitivity of 96% and specificity of 97% for diagnosing abscesses [7]. Accurate assessment of the location, range, and depth of the abscess using MRI can help health care professionals develop more reasonable and effective surgical plans, including selecting the appropriate incision location, accurately positioning the internal opening, and employing the correct drainage method. This comprehensive approach aims to remove the infection as thoroughly as possible, reduce the risk of recurrence and fistula rate, and protect the anal function and quality of life of patients [8].

Diffusion-weighted imaging (DWI) is a variant of conventional MRI based on different rates of water diffusion in different tissues, offering unparalleled sensitivity to water motion within tissue structures [9,10]. Some studies have demonstrated the valuable role of DWI in the diagnosis and differential diagnosis of anal fistula and perianal abscess [11-13]. DWI exhibits high specificity in distinguishing abscesses from inflammatory masses, surpassing traditional MRI [12].

Diffusion tensor imaging (DTI) extends the principles of DWI by modeling water diffusion as a 3D tensor, thereby quantifying both the magnitude and directionality (anisotropy) of diffusion within tissues [14]. Originally developed for neural tractography, DTI has shown promise in musculoskeletal and pelvic floor imaging by revealing microstructural integrity and architectural disruption [15]. A pivotal study by Wang et al [16] demonstrated the feasibility of DTI in evaluating perianal fistula, suggesting that parameters such as fractional anisotropy (FA) and apparent diffusion coefficient could reflect tissue characteristics relevant to surgical planning. However, while this and other emerging studies hint at DTI’s potential, the existing literature remains strikingly sparse regarding its specific application to perianal abscesses. Current evidence is largely extrapolated from fistula research or limited to technical feasibility reports. Critical gaps persist: there is a lack of standardized, quantitative DTI protocols for preoperatively mapping abscess extent, infection infiltration, and precise involvement of the anal sphincter complex and levator ani muscle. Moreover, it remains unclear whether the microstructural metrics provided by DTI correlate with surgical outcomes, such as recurrence rates or postoperative anal function.

### Objectives of the Study

The primary goal of this study is to use DTI to delineate the relationship between the internal opening, the degree of infection and infiltration, the extent of the abscess, and the erosion of perianal tissues and muscles in patients with perianal abscesses. This approach aims to develop an accurate imaging evaluation method for diagnosing the disease, explore the application value of DTI in the diagnosis of perianal abscesses, and improve the accuracy of preoperative evaluation, as well as reduce the damage to the anal sphincter. Ultimately, these efforts aim to reduce the recurrence rate and improve the prognosis and quality of life for patients.

## Methods

### Study Design

**Figure 1.**
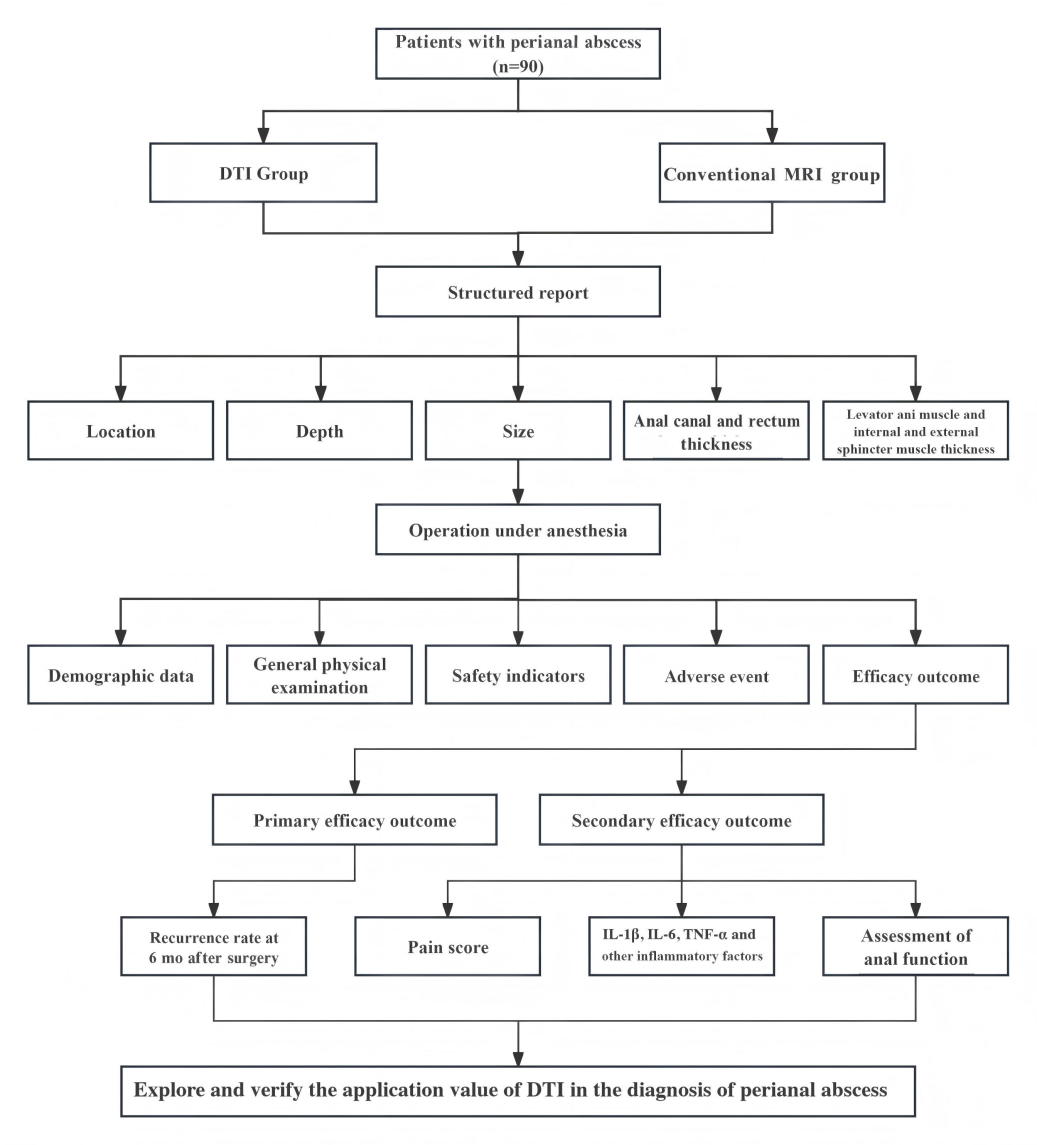
Flowchart of the trial. DTI: diffusion tensor imaging; IL-1β: interleukin-1β; IL-6: interleukin-6; MRI: magnetic resonance imaging; TNF-α: tumor necrosis factor-α.

This study adopts a randomized, controlled, single-blind design to compare the structural characteristics of perianal abscesses and the recurrence rate at 6 months after surgery between the DTI group and the conventional MRI group. Figure 1 illustrates the study flowchart. The schedule of enrollment, interventions, and assessments, in accordance with the SPIRIT (Standard Protocol Items Recommendations for Interventional Trials) guidelines, is summarized in Table 1.

**Table 1.** The Standard Protocol Items Recommendations for Interventional Trials (SPIRIT) schedule table.

	Study period						
	Enrollment	Allocation	Postallocation				Close-out
Timepoint	–t_1_	0	t_1_ (preoperation)	t_2_ (immediately after operation)	t_3_ (the third day after the operation)	t_4_ (the seventh day after the operation)	t_5_ (6 mo after the operation)
Enrollment							
Eligibility screen	✓						
Informed consent	✓						
Allocation		✓					
Interventions							
Diffusion tensor imaging			✓				
Magnetic resonance imaging			✓				
Assessments							
Baseline variables: demographic data, vital signs testing, safety observation indicators			✓				
Recurrence rate							✓
Structured report				✓			
Postoperative pain score					✓	✓	
Detection of inflammatory factors			✓		✓		
Assessment of anal function			✓				✓

### Randomization

In this study, 90 patients with perianal abscess are sequentially numbered from 1 to 90 in order of enrollment. SPSS statistical software is used to generate 90 random 3-digit numbers, which are then sorted in ascending order. The first 45 numbers are assigned to the experimental group, and the remaining 45 to the control group. To implement allocation concealment, the randomization results are sealed in opaque, sequentially numbered envelopes by an independent research coordinator who is not involved in participant recruitment, clinical assessment, or data analysis. Once a patient meets the eligibility criteria and provides written informed consent, the independent coordinator opens the corresponding envelope according to the patient’s number and informs the radiology technician of the group assignment so that the appropriate MRI scanning protocol can be performed. The surgeon then reviews the imaging report generated based on this group assignment before surgery and formulates the surgical plan accordingly.

### Blinding

This study adopts a single-blind design in which participants remain unaware of their group assignments. Surgeons are necessarily unblinded due to the preoperative review of imaging reports. To minimize detection bias, all postoperative outcome assessments, including the evaluation of recurrence, pain scoring, anal function measurement, and inflammatory marker testing, are performed by independent assessors who remain blinded to group allocation throughout the trial.

### Sample Size Calculation

The sample size was calculated based on data from a preliminary pilot study involving 20 patients with perianal abscesses (10 in each preliminary subgroup). Previous studies have reported an overall postoperative recurrence rate for perianal abscess of approximately 11.7% [17]. In the pilot study, the 6-month postoperative recurrence rate was 15% among patients who underwent conventional MRI–guided surgical treatment, while the recurrence rate was 5% in those who received preoperative DTI assessment combined with targeted surgical intervention. Using a 2-sided α level of .05 and a β level of .20, the sample size for the intervention (DTI group) and control (conventional MRI group) was estimated using Power Analysis and Sample Size software (version 11), resulting in N1=N2=41. Considering a 10% potential attrition rate during follow-up, the required sample size for each group was adjusted to 45 (41/0.9), leading to a total enrollment of 90 participants for the formal study.

### Study Population

Patients with perianal abscesses who have surgical indications and meet the inclusion criteria are being recruited from the Anorectal Department of the Shanghai Hospital of Traditional Chinese Medicine between April 2025 and October 2026.

The diagnostic criteria are formulated with reference to the diagnostic part of perianorectal abscess in Chinese Anorectal Diseases [18] and the diagnostic part of perianorectal abscess issued by the State Administration of Traditional Chinese Medicine in 2012 [19]. The systemic symptoms may include chills, high fever, general weakness, and lack of appetite. Additionally, the patient may experience anal swelling, difficulty, or pain when defecating. The presence of hard nodules or lumps around the anal canal, localized pressure or fluctuating sensation, and increased skin temperature. Palpation of the affected area reveals a slightly hard or fluctuating soft mass and a worsening of the pain when pressure is applied. Congested and swollen anal sinus with purulent secretion upon pressing. A puncture examination is conducted, whereby pus is successfully extracted from the most prominent swelling and congestion of the rectal mucosa observed under an anoscope. This procedure proves invaluable in facilitating an accurate diagnosis. A blood routine examination reveals an increase in the total number of white blood cells and neutrophils.

The inclusion criteria are as follows: (1) informed consent and signed informed consent form; (2) meeting the diagnostic criteria of perianal abscess according to both Western medicine and traditional Chinese medicine syndrome of heat and toxin; (3) aged 15 to 80 years; (4) willing to participate and cooperate in completing the required surgical treatment; (5) agree to participate in this experimental study without accepting any other treatment options. In contrast, the exclusion criteria are as follows: (1) patients younger than 15 years old or older than 80 years old; (2) patients who refuse to participate in the study; (3) patients with severe primary diseases of the liver, kidney, hematopoietic system, endocrine system, cardiovascular and cerebrovascular system, nervous system, tuberculosis, vertebral malformation, malignant tumors, or mental illness; (4) patients with a suspected history of abuse of sedative-hypnotics, opioid analgesics, or alcohol. Patients with skin diseases or infectious diseases; (5) individuals with metal implants who cannot undergo MRI; (6) pregnant women, women preparing to become pregnant, or lactating women; (7) individuals who are not suitable or unable to use this treatment for other reasons; (8) individuals with mental disorders, language disorders, motor dysfunction, or psychological diseases.

### Ethical Considerations

The trial is being conducted in accordance with the ethical standards of the Declaration of Helsinki. Ethical approval has been obtained from the Ethics Committee of Shanghai Municipal Hospital of Traditional Chinese Medicine (2023SHL-KYYS-104). Written informed consent is being obtained from all participants after a detailed explanation of the study objectives, procedures, potential risks, and rights (including the right to withdraw at any time without prejudice to clinical treatment), ensuring full comprehension before enrollment. To protect participant privacy and confidentiality, all collected data are being deidentified immediately after acquisition (personal identifiers are replaced with unique study serial numbers) and stored in password-protected electronic databases or locked file cabinets with access restricted to authorized personnel only. No financial compensation is provided to participants for participation in this trial.

### Treatment Plan

Both groups undergo an identical perianal MRI protocol on the same 3.0-T scanner using a 16-channel abdominal phased-array coil, with all participants scanned in the supine position. The sole distinction between the 2 imaging protocols lies in the diffusion-weighted sequence: the control group receives conventional DWI, whereas the intervention group undergoes DTI. Crucially, both groups receive intravenous gadolinium-based contrast agent (GD-DTPA) and subsequent identical postcontrast sequences. All remaining sequences, including T1-weighted, T2-weighted, and fat-suppressed T2-weighted imaging, are standardized across groups.

### Conventional MRI Group

The conventional MRI group underwent imaging using a 3.0-T Philips Ingenia magnetic resonance system equipped with a 16-channel abdominal phased-array coil, with patients scanned in the supine position. The scanning protocol includes the following sequences and parameters. Sagittal fast spin-echo T2-weighted imaging (FSE T2WI) is acquired with an echo time (TE) of 85 ms, repetition time (TR) of 4000 ms, slice thickness of 3.5 mm, slice spacing of 0.5 mm, and matrix size of 416 × 224. Oblique axial FSE T2WI and oblique axial fat-suppressed T2WI are obtained using identical parameters: TE 85 ms, TR 4000 ms, slice thickness 3.5 mm, slice spacing 0.5 mm, and matrix 416 × 224. DWI is performed in the oblique axial plane with a TE of 71 ms, TR of 2000 ms, slice thickness of 5 mm, slice spacing of 1 mm, and a b-value of 800 s/mm². For T1-weighted imaging (T1WI), oblique axial FSE T1WI is acquired with minimum TE, a TR of 400 ms, slice thickness of 3.5 mm, slice spacing of 0.5 mm, and matrix size of 416 × 224. Following intravenous administration of gadolinium-based contrast agent (GD-DTPA), spoiled gradient–recalled echo sequences are performed in oblique axial, oblique coronal, and sagittal orientations, with minimum TE, TR of 200 ms, flip angle of 70°, slice thickness of 3.5 mm, slice spacing of 0.5 mm, and matrix dimensions of 320 × 256.

### The DTI Group

The DTI group is scanned using a 3.0-T Philips Ingenia magnetic resonance imager with a 16-channel abdominal phased array coil, with all participants positioned supine during imaging. The scanning protocol comprises several sequences conducted with specific parameters. Turbo spin-echo T1WI is performed with a TR of 600 ms, TE of 10 ms, a slice thickness of 5 mm, and a total of 20 slices. For transverse axial imaging, both turbo spin-echo T2-weighted and fat-suppressed T2-weighted sequences are acquired using a TR of 1560 ms, TE of 80 ms, slice thickness of 5 mm, and 20 slices. Sagittal fat-suppressed T2WI is carried out with a field of view of 152 × 179 mm, slice thickness of 5 mm, and 20 slices. DTI is conducted using a spin-echo echo-planar imaging sequence with a single excitation in the transverse axis, employing a TR of 3250 ms, TE of 48 ms, 32 diffusion directions, b values of 0 and 600 s/mm², slice thickness of 5 mm, 20 slices, and a total scan time of 5 minutes and 47 seconds.

### Perioperative Management

All surgical procedures in this trial are being performed by surgeons who hold senior professional titles and possess extensive surgical experience. Prior to surgery, preoperative communication is conducted between the medical team and each patient to ensure a clear understanding of the procedural steps and to facilitate cooperation during treatment. Preoperative preparations include an 8-hour fasting and 6-hour fluid restriction period before the procedure. Patients are instructed to take laxatives on the evening prior to surgery and to undergo a cleansing enema on the morning of the operation.

On the day of surgery, perianal hair is shaved and the local area is thoroughly cleansed. Patient positioning—whether lithotomy, prone jackknife, or lateral decubitus—is determined based on the specific location and characteristics of the abscess. Intravenous anesthesia is administered routinely, with general anesthesia reserved for special clinical circumstances.

The surgical approach is decided following a comprehensive evaluation that incorporates the patient’s clinical symptoms, findings from specialized physical examination, and results from auxiliary imaging. The selected intervention is a one-time radical resection of the perianal abscess, aiming to completely address the primary lesion and achieve a curative outcome.

Postoperative care begins on the first day after surgery, with wound dressings being changed twice daily at 8:00 AM and 6:00 PM. During each dressing change, iodophor-soaked cotton balls are used to gently cleanse the pus cavity, wound surface, and any necrotic tissue surrounding the drainage line or tube. Additional wound management interventions, such as cavity irrigation, negative pressure suction, or pad compression, are applied as clinically indicated. In cases where postoperative blood tests suggest infection, intravenous administration of cefuroxime sodium combined with metronidazole is initiated as anti-infective therapy.

### Outcomes

The following data are being systematically collected and analyzed throughout the trial.

#### Demographic and Baseline Data

Data on age, gender, race, height, weight, medical history, and medication history are being recorded for all participants.

#### General Physical Examination

Vital signs are being monitored and documented as part of the initial and follow-up assessments.

#### Safety Monitoring

Safety is being evaluated through a series of routine tests, including complete blood count, urinalysis, stool analysis, liver and kidney function panels, and electrocardiograms.

### Primary Efficacy Outcome

This study assesses the recurrence rate of perianal abscess as the primary endpoint, evaluated at 6 months (±7 d) after surgery. Recurrence is defined by the reappearance of clinical symptoms (eg, perianal pain, swelling, or purulent discharge) along with confirmation on a follow-up MRI scan—specifically the DTI sequence—of a new or persistent fluid collection suggestive of an abscess. For the purpose of this primary outcome, the presence of a newly developed anal fistula also constitutes a recurrence event. All assessments for recurrence are performed by 2 independent, experienced radiologists. These radiologists are blinded to the patient’s group allocation and baseline imaging findings. Any discrepancy between their assessments is resolved either by consensus or by a third senior radiologist.

### Secondary Efficacy Outcomes

#### Structured Imaging Report

The structured report (see Table S1 in Multimedia Appendix 1) is completed following the surgical procedure and serves as a synthesis of direct intraoperative findings and preoperative DTI results. During surgery, an experienced colorectal surgeon meticulously explores the lesion and documents the key anatomical conditions. These surgical observations are then integrated with the preoperative DTI interpretations provided by a senior radiologist to finalize the report collaboratively. It is important to clarify that this report is compiled postintervention and therefore does not inform real-time intraoperative decisions. To ensure consistency, all participating surgeons and radiologists received standardized training on the reporting items and definitions prior to the study. To assess inter-rater reliability, a subset of the completed reports will undergo independent review by a second surgeon who remains blinded to the initial assessment.

#### Postoperative Pain Score

Pain scores: The visual analog scale is being used to measure postoperative pain on days 3 and 7 [20]. Participants are asked to draw a horizontal line on a piece of paper, with 0 at one end representing no pain and 10 at the other end representing severe pain. Participants then mark a point on the line corresponding to the intensity of their pain sensation. The distance from point 0 to the marked point is measured to determine the pain intensity score.

#### Detection of Inflammatory Factors

Peripheral blood samples are collected to measure the levels of interleukin (IL)-1β, IL-6, tumor necrosis factor-α, IL-2, IL-4, IL-5, IL-8, IL-10, IL-12p70, IL-17A, interferon-γ, and interferon-α2. All sample processing and testing are conducted in strict accordance with the standardized operating procedures of the hospital’s clinical laboratory. Antibody chips are being employed to covalently bind highly specific inflammatory factor-capture antibodies onto chemically modified solid-phase chip substrates. Signal amplification is achieved through the use of high-affinity paired secondary antibodies, enabling the effective detection of trace cytokines. Furthermore, a standard curve is constructed using the detection signals of gradient dilutions of standards, allowing for accurate quantitative detection of inflammatory factors in human samples by standard curve fitting. All laboratory procedures are carried out by trained staff who are blinded to group assignments. Intra-assay and inter-assay coefficients of variation are consistently controlled below 10%, ensuring the reliability of the analytical results.

#### Assessment of Anal Function

The Wexner Anal Incontinence Score [21] is being used to assess the patient’s anal function before and 6 months after surgery. This score evaluates anal function and records incontinence in terms of dry stools, loose stools, gas, need for pads, and lifestyle changes (See Table S2 in Multimedia Appendix 1).

Assessments are conducted by trained research assistants who are blinded to group allocation and baseline scores. All assessors received standardized training prior to study initiation to ensure consistent administration and interpretation of the Wexner scoring system. At each assessment time point, the Wexner questionnaire is administered via a structured interview format. To further mitigate potential assessment bias, baseline scores are recorded prior to randomization and will be incorporated as covariates in the statistical models used to compare postoperative functional outcomes between the 2 groups.

### Follow-Up Plan

To ensure complete data collection, all participants will be contacted according to a standardized follow-up schedule. Automated text message reminders will be sent 2 days before each scheduled visit (postoperative days 3 and 7, and month 6), followed by a confirmatory telephone call 1 day prior. For the 6-month assessment, participants who are unable to attend an in-person will be offered a structured telephone interview to evaluate symptoms and functional status; if recurrence is suspected during the interview, an urgent in-person clinical evaluation, including DTI reassessment, will be arranged.

### Adverse Events Monitoring and Reporting

Adverse events (AEs) are defined as any unfavorable medical occurrence experienced by trial participants, irrespective of causal relationship to the study intervention. This includes newly emerging symptoms, clinically relevant abnormal laboratory findings, or exacerbations of preexisting medical conditions. A serious adverse event (SAE) is defined as any AE that fulfills at least one of the following criteria: results in death, is life-threatening, necessitates inpatient hospitalization or prolongation of existing hospitalization, results in persistent or significant disability/incapacity, or constitutes a congenital anomaly or birth defect.

All AEs will be graded according to the Common Terminology Criteria for Adverse Events, version 5.0, as follows: grade 1 (mild: asymptomatic or mild symptoms, no intervention indicated); grade 2 (moderate: minimal intervention indicated, interferes with activities of daily living); grade 3 (severe: medically significant but not immediately life-threatening, hospitalization indicated); grade 4 (life-threatening: urgent intervention required); and grade 5 (death). All AEs are systematically recorded at each study visit and during scheduled follow-up interactions. Nonserious AEs are documented in the case report forms and will be summarized in the final study report. SAEs must be reported to the principal investigator and the Institutional Ethics Committee within 24 hours of awareness, with a comprehensive written report submitted within 7 calendar days, followed by continuous documentation until event resolution or stabilization.

An independent Data and Safety Monitoring Board has been established to conduct periodic reviews of SAEs, interim safety data, and overall trial safety. The Ethics Committee maintains the authority to request supplementary information, recommend protocol modifications, or suspend the study based on safety evaluations. All investigators and study personnel have completed standardized training in AE/SAE identification, documentation, and reporting procedures prior to study initiation.

### Data Management and Quality Assurance

All study data will be managed through an electronic data capture system equipped with built-in logic checks and mandatory field validation to ensure data integrity, accuracy, and consistency. After entry, data will undergo dual independent verification, with systematic reviews conducted by data managers to identify outliers and address missing values. Data and Safety Monitoring Board will conduct periodic assessments of safety data, protocol compliance, and overall data quality. All study personnel have completed standardized training to maintain consistency and procedural rigor throughout data collection and evaluation processes.

### Statistical Plan

All analyses are prespecified. The primary analysis will follow the intention-to-treat principle. For the primary outcome, the 6-month recurrence rate will be analyzed using binary logistic regression, with results reported as an adjusted odds ratio and 95% CI. Any clinically relevant baseline imbalances will be included as covariates, and missing recurrence data will be handled using multiple imputation. Secondary outcomes will be analyzed as follows: categorical items from the structured report will be compared using chi-square test or Fisher exact test; continuous structured report items will be compared using independent samples *t* tests or Mann-Whitney *U* tests, depending on normality; postoperative visual analog scale pain scores will be evaluated with a linear mixed model to assess group, time, and interaction effects, with results expressed as estimated marginal means and mean differences with 95% CI; and inflammatory markers and Wexner incontinence scores will be compared at follow-up using *t* tests or Mann-Whitney *U* tests. To control for type I error across secondary outcomes, the false discovery rate procedure will be applied. Prespecified sensitivity analyses using the per-protocol population and subgroup analyses stratified by abscess complexity will also be performed. All statistical analyses will be conducted using SPSS software (version 22.0), and statistical significance will be defined as a 2-sided *P* value < .05 .

### Management of Protocol Amendments

Any proposed amendment to the study protocol must be submitted for review and approval by the Ethics Committee prior to implementation. Approved amendments will be promptly reflected in the public clinical trial registry, and all investigators and relevant stakeholders will be formally informed. No protocol amendment shall adversely affect the rights, safety, or well-being of already enrolled participants. Any modification deemed to be of potential benefit to participants may only be implemented with Ethics Committee approval and, where applicable, following informed consent from the affected participants.

### Management of Participant Withdrawal

Participants retain the right to withdraw from the study at any stage without condition, and such withdrawal will not affect their future medical care. If a participant withdraws, data collected prior to withdrawal may be retained for use in the intention-to-treat analysis, provided that the participant has given consent for such use. If consent is not provided, the participant’s data will be deleted and excluded from the final analysis. The reason for withdrawal will be documented and reported. In cases where withdrawal is due to an AE, appropriate follow-up will be conducted until the event is resolved or stabilized.

## Results

The trial was registered on January 10, 2025, with the International Traditional Medicine Clinical Trial Registry (ITMCTR2025000068). The study obtained ethical approval in 2023. Participant recruitment began in April 2025 and is expected to continue until October 2026. Completion of the 6-month postoperative end point follow-up is projected by April 2027. Preliminary data analysis is planned for the second quarter of 2027, and the full study report anticipated to be submitted and published before the end of 2027. As of January 2026, a total of 37 participants had been enrolled. Results will be submitted for publication in a peer reviewed international journal. Findings will also be presented at relevant national and international conferences.

## Discussion

This study is designed to test the hypothesis that preoperative DTI, compared with conventional MRI, can provide superior visualization of perianal abscess extent and muscular involvement, thereby guiding more precise surgical excision and leading to a lower 6-month recurrence rate.

Currently, there is only 1 comparative study between DTI and MRI for perianal fistula [16], and no specific studies have been reported on the location depth, size, thickness of anal canal and rectum, and thickness of the levator ani muscle and internal and external sphincter of perianal abscesses. This protocol builds upon the feasibility shown in fistula imaging but shifts the focus to preoperative mapping of abscess morphology and infiltration—a critical gap in current clinical practice.

Perianal abscess is a common disease in the anorectal department, and surgery remains the most effective and complete cure treatment [22]. However, the relationship between the extent of abscess involvement and surrounding inflammatory infiltration is not determined before the operation, which can increase the risk of postoperative recurrence [23]. Although conventional MRI sequences have some diagnostic value in determining the extent of disease, they have limitations in distinguishing abscesses from inflammatory masses [24,25]. Therefore, a contrast agent that shows the characteristic edge enhancement of an abscess is often used in combination. However, there are increasing concerns regarding contrast injection, especially in patients with renal insufficiency, and the association between contrast exposure and nephrogenic systemic fibrosis has been emphasized [26]. Therefore, the search for alternative imaging techniques that do not use contrast agents has been an important topic, especially for high-risk patients, while ensuring that diagnostic value is maintained. DTI can clearly show the degree of infection infiltration, the extent of the abscess, and the relationship between the erosion of tissue and muscle around the pelvic floor [27,28]. This makes DTI essential for reducing the chance of recurrence. In addition, the fractional anisotropy and apparent diffusion coefficient values of the DTI parameters may play a role in assessing the extent and activity of the perianal abscess.

In this trial, 90 participants are being enrolled and divided into 2 groups, a DTI group and a conventional MR group, based on randomization performed before enrollment. “The primary variable, recurrence rate, and secondary variables, including structured reports, pain scores on postoperative days 3 and 7, peripheral inflammatory factors, and assessment of anal function, are being assessed at 6 months after surgery.” To ensure accurate and specific information, a structured report on the extent of perianal abscess infection based on DTI images is being prepared by both anorectal physicians and radiologists. Surgeons experienced in examining and operating on the lesions can provide valuable insights into the actual situation of the patient during surgery. By using a structured report, we aim to determine whether the test under consideration is effective and which of the 2 tests is more effective.

Although the recurrence rate of perianal abscesses after surgery is influenced by numerous factors, there is no doubt that the accuracy of the examination can greatly improve the success rate of surgery [29]. Currently, surgical treatment is the main method for perianorectal abscesses in clinical medicine. However, the location, number, specific shape of the abscess cavity, and its relationship with surrounding muscles of perianorectal abscess have a direct impact on the choice of surgical methods, which in turn will influence its prognosis [30]. Therefore, it is very important to pay attention to the preoperative diagnosis of such patients for the implementation of targeted and effective surgical treatment. Therefore, by using DTI MRI imaging sequences, we can scan the internal opening, the degree of infection and infiltration, the extent of abscess, and the relationship between the erosion of perianal tissues and muscles, to explore a new imaging method for the evaluation and diagnosis of perianal abscess. This approach can explore the application value of DTI in the diagnosis of perianal abscess. It can improve the accuracy of preoperative evaluation, reduce damage to the anal sphincter, reduce the recurrence rate, and improve the quality of life of patients.

DTI brings additional challenges to the diagnosis of perianal abscess. First, there is no uniform standard for measuring the thickness and ratio of the relevant muscles before surgery for perianal abscess, as well as the location, depth, and size of the abscess. To address this issue, relevant researchers will be trained by the director of radiology, relevant literature will also be reviewed to formulate unified standards. Second, monitoring MRI and operating room schedules is needed to identify potential study patients and appoint someone to manage the imaging data. Despite the challenges of such data collection efforts, many of the challenges of integrating research into clinical workflows can be overcome. DTI is a useful clinical tool for surgical planning and intraoperative guidance. By using DTI technology, we can gain a deeper understanding of the relationship between the internal orifice, the degree of infection infiltration, the extent of abscess, and the erosion of perianal tissues and muscles. These data may provide a basis for surgical treatment, and we hope that it will form a new technical standard for the diagnosis of perianal abscesses in the future.

It is hoped that the dataset being collected in this study will represent the objective parameters of the perianal muscular tissues and also effectively respond to the patient population we are eventually serve. As expected, due to regional ethnic differences, more data will need to be collected in the future, but it is expected that based on this study, the draft technical standards of DTI for perianal abscess diseases will be established, and new techniques for diagnosis and evaluation of perianal abscess will be developed and formed. This will serve the surrounding hospitals and reduce the postoperative recurrence rate and reoperation rate of perianal abscess patients.

## Supplementary material

10.2196/83449 Multimedia Appendix 1 Supplementary tables of the DTI structural report of perianal abscess and the Wexner anal incontinence score.

## Abbreviations

AE: adverse event
DTI: diffusion tensor imaging
DWI: diffusion-weighted imaging
FSE: fast spin-echo
GD-DTPA: Gadolinium-Diethylenetriamine Pentaacetate
IL: interleukin
MRI: magnetic resonance imaging
SAE: serious adverse event
SPIRIT: Standard Protocol Items Recommendations for Interventional Trials
T1WI: T1-weighted imaging
T2WI: T2-weighted imaging
TE: echo time
TR: repetition time

## References

[1] Sahnan K, Adegbola SO, Tozer PJ, Watfah J, Phillips RK (2017). Perianal abscess. BMJ.

[2] Wang D, Yang G, Qiu J (2014). Risk factors for anal fistula: a case-control study. Tech Coloproctol.

[3] Gaertner WB, Burgess PL, Davids JS (2022). The American Society of Colon and Rectal Surgeons Clinical Practice Guidelines for the management of anorectal abscess, fistula-in-ano, and rectovaginal fistula. Dis Colon Rectum.

[4] Vogel JD, Johnson EK, Morris AM (2016). Clinical practice guideline for the management of anorectal abscess, fistula-in-ano, and rectovaginal fistula. Dis Colon Rectum.

[5] Amato A, Bottini C, De Nardi P (2020). Evaluation and management of perianal abscess and anal fistula: SICCR position statement. Tech Coloproctol.

[6] Villa C, Pompili G, Franceschelli G (2012). Role of magnetic resonance imaging in evaluation of the activity of perianal Crohn’s disease. Eur J Radiol.

[7] Beets-Tan RG, Beets GL, van der Hoop AG (2001). Preoperative MR imaging of anal fistulas: does it really help the surgeon?. Radiology.

[8] Dong S, Chen B, Zhang J (2023). Study on the factors influencing the prognosis after perianal abscess surgery. BMC Gastroenterol.

[9] Gullo RL, Partridge SC, Shin HJ, Thakur SB, Pinker K (2024). Update on DWI for breast cancer diagnosis and treatment monitoring. AJR Am J Roentgenol.

[10] Alyami AS (2024). Imaging of ulcerative colitis: the role of diffusion-weighted magnetic resonance imaging. J Clin Med.

[11] Hori M, Oto A, Orrin S, Suzuki K, Baron RL (2009). Diffusion‐weighted MRI: a new tool for the diagnosis of fistula in ano. J Magn Reson Imaging.

[12] Dohan A, Eveno C, Oprea R (2014). Diffusion-weighted MR imaging for the diagnosis of abscess complicating fistula-in-ano: preliminary experience. Eur Radiol.

[13] Bakan S, Olgun DC, Kandemirli SG (2015). Perianal fistula with and without abscess: assessment of fistula activity using diffusion-weighted magnetic resonance imaging. Iran J Radiol.

[14] Tae WS, Ham BJ, Pyun SB, Kang SH, Kim BJ (2018). Current clinical applications of diffusion-tensor imaging in neurological disorders. J Clin Neurol.

[15] Zijta FM, Froeling M, Nederveen AJ, Stoker J (2013). Diffusion tensor imaging and fiber tractography for the visualization of the female pelvic floor. Clin Anat.

[16] Wang Y, Gu C, Huo Y (2018). Diffusion tensor imaging for evaluating perianal fistula: feasibility study. Medicine (Baltimore).

[17] JS HU, WANG W, LM SUN (2023). Analysis of influencing factors of anal fistula formation after perianal abscess operation with different operation methods. Chin J Surg Integr Trad West Med.

[18] Huang NJ (1996). Chinese Journal of Colorectal Diseases [Book in Chinese].

[19] State Administration of Traditional Chinese Medicine (SACM) (1994). Traditional Chinese Medicine Disease and Syndrome Diagnosis and Treatment Standards [Book in Chinese].

[20] Kelly AM (2001). The minimum clinically significant difference in visual analogue scale pain score does not differ with severity of pain. Emerg Med J.

[21] Agachan F, Chen T, Pfeifer J, Reissman P, Wexner SD (1996). A constipation scoring system to simplify evaluation and management of constipated patients. Dis Colon Rectum.

[22] Qiheng L, Maonan W, Siqi L, Tao M (2024). Progress of Chinese and Western medicine treatment of perianal abscess [Article in Chinese]. Jilin Med J.

[23] Amato A, Bottini C, De Nardi P (2015). Evaluation and management of perianal abscess and anal fistula: a consensus statement developed by the Italian Society of Colorectal Surgery (SICCR). Tech Coloproctol.

[24] Hopkins KL, Li KC, Bergman G (1995). Gadolinium-DTPA-enhanced magnetic resonance imaging of musculoskeletal infectious processes. Skeletal Radiol.

[25] Kan JH, Young RS, Yu C, Hernanz-Schulman M (2010). Clinical impact of gadolinium in the MRI diagnosis of musculoskeletal infection in children. Pediatr Radiol.

[26] Daftari Besheli L, Aran S, Shaqdan K, Kay J, Abujudeh H (2014). Current status of nephrogenic systemic fibrosis. Clin Radiol.

[27] Zijta FM, Froeling M, van der Paardt MP (2011). Feasibility of diffusion tensor imaging (DTI) with fibre tractography of the normal female pelvic floor. Eur Radiol.

[28] Zijta FM, Lakeman MME, Froeling M (2012). Evaluation of the female pelvic floor in pelvic organ prolapse using 3.0-Tesla diffusion tensor imaging and fibre tractography. Eur Radiol.

[29] Chun CW, Jung JY, Baik JS, Jee WH, Kim SK, Shin SH (2018). Detection of soft-tissue abscess: comparison of diffusion-weighted imaging to contrast-enhanced MRI. J Magn Reson Imaging.

[30] van Koperen PJ, Wind J, Bemelman WA, Bakx R, Reitsma JB, Slors JFM (2008). Long-term functional outcome and risk factors for recurrence after surgical treatment for low and high perianal fistulas of cryptoglandular origin. Dis Colon Rectum.

