# Supplementary material for: Diffusion Tensor Imaging in the Diagnosis of Perianal Abscess: Protocol for a Single-Blind Randomized Controlled Trial
Source: JMIR Res Protoc. 2026 Feb 25;15:e83449. doi: 10.2196/83449 (PMC12935422; doi:10.2196/83449)
Supplement: Multimedia Appendix 1 [file resprot-v15-e83449-s001.docx]

**Table S1.** DTI structural report of perianal abscess.

| Item | Concrete Result |
| --- | --- |
| Location of Abscess |  |
| Depth of Abscess |  |
| Extent of Involvement of Levator Ani Muscle |  |
| Relationship with Anal Sphincter Complex |  |
| Presence of Lesions Above Levator Ani Muscle |  |
| Thickness of Anal Canal |  |
| Thickness of Rectum |  |
| The thickness of Levator Ani Muscle |  |
| Thickness of External Sphincter |  |
| Thickness of Internal Sphincter |  |
| ADC Value of Perianal Abscess |  |
| FA Value of Perianal Abscess |  |

**Table S2.** The Wexner anal incontinence score.

| Incontinence situation | Frequency | | | | | Score |
| --- | --- | --- | --- | --- | --- | --- |
|  | Never | Rarely | Sometimes | Weekly | Daily |  |
| Solid stool | 0 | 1 | 2 | 3 | 4 |  |
| Liquid stool | 0 | 1 | 2 | 3 | 4 |  |
| Gas | 0 | 1 | 2 | 3 | 4 |  |
| Need to wear a pad | 0 | 1 | 2 | 3 | 4 |  |
| Lifestyle alteration | 0 | 1 | 2 | 3 | 4 |  |
| Total score ( ) | | | | | | |
